# Lysosomal function in neurodegenerative diseases

**DOI:** 10.1186/1750-1326-7-S1-O2

**Published:** 2012-02-07

**Authors:** Jianhua Zhang

**Affiliations:** 1 Center for Free Radical Biology, Department of Pathology, University of Alabama at Birmingham, Birmingham VA Medical Center, Birmingham, AL 35294, USA

## 

Protein aggregation, mitochondrial deficits, oxidative and autophagic stress are prominent pathologies in neurodegenerative diseases. Our laboratory has a long term interest in the regulation of autophagy-lysosomal activities in neurodegeneration. We previously found that deficiency in the lysosomal cathepsin D in a mouse knockout model led to accumulation of the key protein in Parkinson’s disease, α-synuclein. Overexpression of cathepsin D in human neuroblastoma cells as well as worms reduced α-synuclein aggregation and toxicity. Recent work found that enhanced expression of lysosomal cathepsin D reduced both full-length and fragmented huntingtin in transfected HEK cells, and protected against mutant huntingtin toxicity in primary neurons. Furthermore, as mitochondria are the major generators and targets of reactive species, and oxidative stress has been found in a wide range of neurodegenerative diseases, investigating the source and nature of oxidative stress in cathepsin deficient brains are of significant interest. It has been previously shown that α-synuclein modification by S-nitrosylation enhances its aggregation propensity. Furthermore, microglial produced nitrous oxide (NO) from an increase in inducible nitrous oxide synthase (iNOS) underlies some of the brain pathologies in CD knockout mice. Given that other lysosomal storage conditions have demonstrated mitochondrial involvement and that oxidative and nitrative changes have been associated with CD deficiency, we studied the effects of CD deficiency on mitochondrial morphology and function with the intent to understand how this lysosomal enzyme play a role in maintaining cellular bioenergetics and management of cellular oxidative states. We have found that although there are no changes in total mitochondrial biomass due to CD deficiency, there are enlarged mitochondria, and deficits in functional parameters of the electron transport chain. The changes demonstrated in this study contribute to the understanding of how mitochondrial/lysosomal interplay affects the pathogenesis of neurodegenerative diseases.

